# Pain-Induced Pulsograph Changes in Patients with Primary Dysmenorrhea: A Pilot Study

**DOI:** 10.1155/2015/385136

**Published:** 2015-09-30

**Authors:** Wan-hong Chen, Yan Zhao, Chang-chun Zeng, Dao-ning Zhang, Yan-ping Wang, Ling Tang, Xiao-mei Zhang, Tian-fang Wang

**Affiliations:** ^1^Department of Diagnostics of Traditional Chinese Medicine, School of Preclinical Medicine, Beijing University of Chinese Medicine, Beijing 100029, China; ^2^School of Basic Medicine Sciences, Guilin Medical University, Guangxi, Guilin 541004, China; ^3^School of Acupuncture and Moxibustion, Beijing University of Chinese Medicine, Beijing 100029, China; ^4^Department of Gynecology, Dongzhimen Hospital, Beijing University of Chinese Medicine, Beijing 100700, China; ^5^School of Humanities, Beijing University of Chinese Medicine, Beijing 100029, China

## Abstract

*Objectives*. To investigate changes in pulsograph caused by pain in primary dysmenorrhea (PD) patients. *Methods*. Pulsograph and pain level of PD patients were detected using electropulsograph and Visual-Analogue Scale (VAS), respectively, at four time points, 7–10 days before menstruation (*T*0), maximal pain during menstruation (*T*1), immediately after acupuncture analgesia (*T*2), and 30 mins after acupuncture analgesia (*T*3). Parameters (*t*, *h*, *w*) and normalized time parameters (*t*′) of pulsograph were analyzed. *Results*. VAS pain scores decreased from 6.40 ± 1.13 at *T*1 to 0.70 ± 0.75 at *T*2 to 0.11 ± 0.32 at *T*3 (*P* < 0.001 and 0.001). At *T*1, compared with those at *T*0, *w*1, *h*3, and *h*4 significantly increased (*P* < 0.01), and *t*2, *t*2′, *t*3′, and *h*(d) significantly decreased (*P* < 0.01, 0.001, 0.05, and 0.001). At *T*2, compared with those at *T*1, *t*1, *w*1, *w*2, *h*2, *h*3, *t*1′, and *t*4′ significantly decreased (*P* < 0.05, 0.01, 0.01, 0.001, 0.01, 0.001, and 0.05), and *h*(d) significantly increased (*P* < 0.001). There was no difference between *T*2 and *T*3. *Conclusions*. There are almost opposite changing trends in pulsographic parameters when pain occurs and when it is relieved in PD patients.

## 1. Introduction

Pulse assessment is an assessing method used in Traditional Chinese Medicine (TCM). The main site for pulse feeling is the radial artery located in the inner side of radial styloid process at wrist, also called* cunkou* in Chinese. The* cunkou* at each hand is divided into the following three portions, respectively. The* guan* position is in the middle part of* cunkou*, just near the radial styloid process,* cun* portion between the wrist crease and* guan* portion, and* chi* portion on the other side of* guan* portion. By feeling the pulse condition, TCM clinicians may know the unique TCM pattern status of the human body [1].

*Diagnostics of TCM*, a textbook widely used at TCM colleges and universities which is about how to examine illness and recognize syndrome in TCM, recorded that pain is often accompanied by wiry pulse, tight pulse, or tremulous pulse [2]. Clinical researcher also found that the wiry pulse is the most common pulse (62.9%) in patients with pain like headaches and chest pain. Moreover, tight pulse, deep pulse, and thready pulse are also seen in those patients [3].

In view of the subjectivity of clinicians' pulse feeling, increasing interest has been focused on pulse apparatus where sensors are developed to acquire pulse signals. Pulse apparatuses are exploited to analyze pulse conditions [4]. Recently, an increasing number of studies on pulsograph have been done in clinical research [5–7]. However, only a few studies have focused on the pulsograph of patients suffering pain. One of the studies' results showed that there were significant differences in *h*1, *h*3, *h*4, and *h*5 of pulsograph during menstruation between different TCM patterns of 148 PD patients [8, 9].

Pain affects pulse conditions, giving rise to pulse changes. Accordingly, we designed the current research to investigate the pain-induced changes in pulsograph parameters. Primary dysmenorrhea (PD) is a common disease in young women. In view of its regular episode and quick relieving after treatment we chose PD representing pain to provide a basis for further study on pulse condition associated with pain in TCM.

## 2. Methods

### 2.1. Subjects

From April 2014 to May 2014, college students in Beijing University of Chinese Medicine who were aged 18 to 30 years with dysmenorrhea with pain level reaching 5 scores on a Visual-Analogue Scale (VAS) within the previous 3 regular menstrual cycles were recruited. PD patients who were diagnosed by the follow-up clinical examinations were invited to participate in the study. The diagnosis was made according to Primary Dysmenorrhea Clinical Guidelines made by Society of Obstetricians and Gynecologists of Canada (SOGC) in 2005. Accordingly, all patients had periodical pain in the lower abdomen before or during menstruation with normal pelvic examination and ultrasound [10].

Since unnatural changes of body may impact pulse [2], we excluded patients who had a history of injury or who caught a cold within the previous week, or who were complicated with any diseases of the heart, liver, kidney, and peripheral nerves, and so forth. For convenient detection, we excluded patients with physiologic variations or skin damage in left* cunkou*.

### 2.2. Observing Time Points

We observed PD patients at 4 time points: 7–10 days before menstruation (*T*0), maximal pain during menstruation (*T*1), immediately after acupuncture analgesia (*T*2), and 30 mins after acupuncture analgesia (*T*3).

### 2.3. Pain Measurement

Pain intensity was assessed at *T*0, *T*1, *T*2, and *T*3 by using a 10 cm VAS, a scale presented as a horizontal row of equidistant numbers from 0 to 10, with ratings that ranged from “no pain” at 0 to “pain as bad as you can imagine” at 10 [11].

### 2.4. Pulse Apparatus and Gathering of Pulsograph

**Figure 1:**
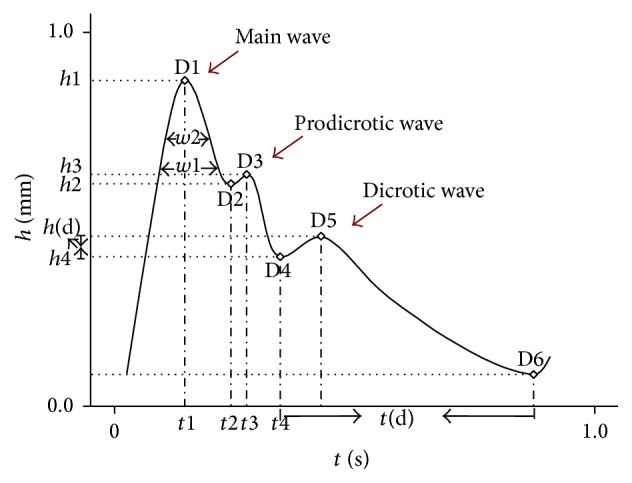
Pulsograph and pulsographic parameters.

In this study pulsograph was detected by DS01-C electropulsograph where baroreceptors were developed to acquire pulse signals produced by Shanghai Daosheng Medical Limited Company. The pulse signals were converted into pulsograph through a computer system. The characteristics extraction and analysis of pulsograph were done by using the time-domain technique [12]. As shown in Figure 1, a standard pulsograph is composed of main wave, prodicrotic wave, and dicrotic wave. The main wave and prodicrotic wave correspond to systole while the dicrotic wave corresponds to diastole. The analyzed pulsographic parameters in this study were as below: time and amplitude of the peak of the main wave (*t*1, *h*1) and gorge of main wave (*t*2, *h*2) and peak of the prodicrotic wave (*t*3, *h*3) and dicrotic notch (*t*4, *h*4), width of the upper 1/3 (*w*1) and 1/5 (*w*2) of the main wave, the time of dicrotic wave (*t*(d)), and amplitude of the peak of dicrotic wave (*h*(d)).

*Cunkou* is the main position for pulse feeling in TCM and also is the only part which electropulsograph can detect. TCM holds that left* cunkou* can better show the body situation of women because it mainly reflects the situation of heart, liver, and kidney. In addition, left* cunkou* can avoid the influence on the pulse by different motion styles in right-handed individuals. Electropulsograph only has one probe and* guan* position is more easily to be accurately detected. So pulsograph of* guan* position of left* cunkou* was detected in this study.

Pulsograph was detected at *T*0, *T*1, *T*2, and *T*3. Specific detection method was as follows: patients were required in supine position, with forearm stretched, wrist straight, palm up, and wrist at the same level with heart. Trained experimenter fixed the probe of collector at the* guan* position of left* cunkou*. The computer would collect pulsographs of different pressures when the patients were relaxed and breathed naturally and then select the best pulsograph automatically for analysis.

### 2.5. Acupuncture Analgesia

Previous researches prove that acupuncture has instant analgesic effect [13] and is of high efficiency in primary dysmenorrhea [14]. Besides, considering the acceptance of acupuncture for subjects in this study and side effects of painkillers [15] acupuncture was chosen to alleviate primary dysmenorrhea.

Acupuncture was initiated at *T*1. Selected acupoints included Sanyinjiao (SP6), Diji (SP8), and Yinlingquan (SP9), on both sides [16]. Manipulation is as follows: the patient was in a supine position with local skin exposed. After routine disinfection with 75% alcohol cotton balls on the local skin, disposable acupuncture needles (40 mm × 0.30 mm) were then inserted at SP6, SP8, and SP9 perpendicularly in the depth of about 1 cm. Lift, thrust, and twist gently till the patients had sensations of soreness and distension. All the needles were retained for 30 mins.

### 2.6. Statistical Analysis

Measurement data were presented as mean ± standard. The measurement data were analyzed by *t*-test. Differences with *P* < 0.05 were considered statistically significant. The statistical evaluation was performed by using the statistical software package SPSS 17.0.

## 3. Results

### 3.1. Study Population

30 patients with PD were enrolled with a mean age of 23 years (range, 19 to 29), and with an average suffering time of 7 years (range, 2 to 15). All the patients had provided written informed consent before completion of the experiment between May and August 2014.

### 3.2. VAS Pain Scores

**Figure 2:**
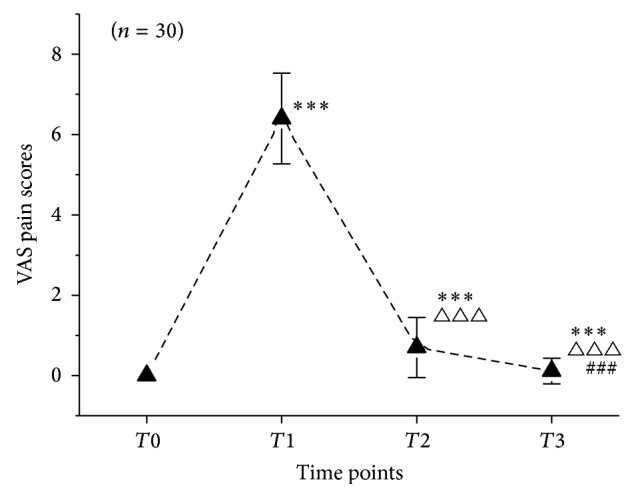
VAS pain scores of 30 PD patients at *T*0, *T*1, *T*2, and *T*3. Notes: *T*0 means 7–10 days before menstruation, *T*1 means maximal pain (VAS pain scores ≥ 5) during menstruation, *T*2 means immediately after acupuncture analgesia, and *T*3 means 30 mins after acupuncture analgesia. ^*∗∗∗*^ *P* < 0.001, compared with *T*0; ^△△△^ *P* < 0.001, compared with *T*1; ^###^ *P* < 0.001, compared with *T*2.

As shown in Figure 2, 30 enrolled PD patients showed no pain at *T*0. At *T*1, patients experienced the maximal pain, and the reading on the VAS increased drastically to 6.40 ± 1.13. With acupuncture analgesia, compared with the reading at *T*1, VAS pain scores significantly decreased to 0.70 ± 0.75 at *T*2 (*P* < 0.001). After acupuncture analgesia, the pain scores at *T*3 significantly decreased to 0.11 ± 0.32 (*P* < 0.001), compared with that at *T*2.

### 3.3. Changes in Pulsograph Parameters during Systole in PD Patients

**Figure 3:**
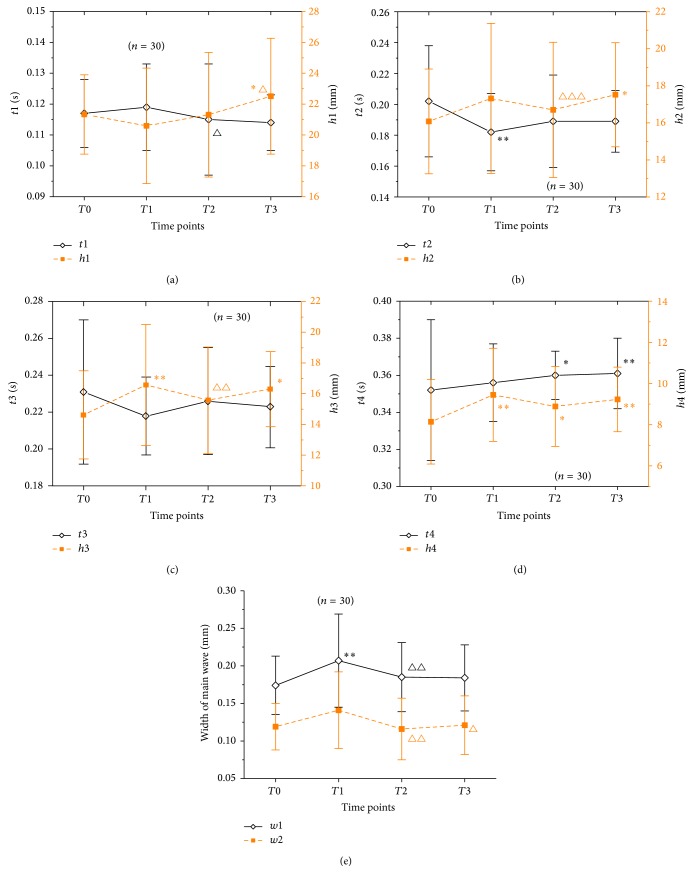
Pulsograph parameters during systole in 30 PD patients at *T*0, *T*1, *T*2, and *T*3. Notes: (a), (b), (c), and (d) were the changes in the time and amplitude of D1, D2, D3, and D4. (e) was the changes in the width of the upper 1/3 (*w*1) and 1/5 (*w*2) of the main wave. ^*∗∗*^ *P* < 0.01 and ^*∗*^ *P* < 0.05, compared with *T*0; ^△△△^ *P* < 0.001, ^△△^ *P* < 0.01, and ^△^ *P* < 0.05, compared with *T*1.

From *T*0 to *T*1, time of peak of the main wave (*t*1) increased (*P* > 0.05) and amplitude of peak of main wave (*h*1) decreased (*P* > 0.05) (Figure 3(a)). Time of gorge of the main wave (*t*2) decreased significantly (*P* < 0.05) and amplitude of gorge of the main wave (*h*2) increased (*P* > 0.05) (Figure 3(b)). Time of peak of the prodicrotic wave (*t*3) decreased (*P* > 0.05) and amplitude of peak of prodicrotic wave (*h*3) increased significantly (*P* < 0.01) (Figure 3(c)). Time of dicrotic notch (*t*4) increased (*P* > 0.05) and amplitude of dicrotic notch (*h*4) increased significantly (*P* < 0.01) (Figure 3(d)). Widths of the upper 1/3 (*w*1) and 1/5 (*w*2) of main wave both increased (*P* < 0.01 and *P* > 0.05) (Figure 3(e)).

From *T*1 to *T*2 to *T*3, *t*1 decreased gradually and there was statistically significant difference between *T*1 and *T*2 (*P* < 0.05). Meanwhile, *h*1 increased gradually and was significantly higher at *T*3 than at *T*1 (*P* < 0.05) (Figure 3(a)). *t*2 increased (*P* > 0.05), while *h*2 significantly decreased (*P* < 0.05) at first and then increased (*P* > 0.05) (Figure 3(b)). *t*3 increased at first (*P* > 0.05) and then decreased (*P* > 0.05), while *h*3 significantly decreased (*P* < 0.001) at first and then decreased (*P* > 0.05) (Figure 3(c)). *t*4 increased (*P* > 0.05), while *h*4 decreased (*P* > 0.05) at first and then increased (*P* > 0.05) (Figure 3(d)). Both *w*1 and *w*2 demonstrated significant reductions at first (*P* < 0.01) and then had no significant changes (*P* > 0.05) (Figure 3(e)).

### 3.4. Changes in Pulsographic Parameters during Diastole in PD Patients

**Figure 4:**
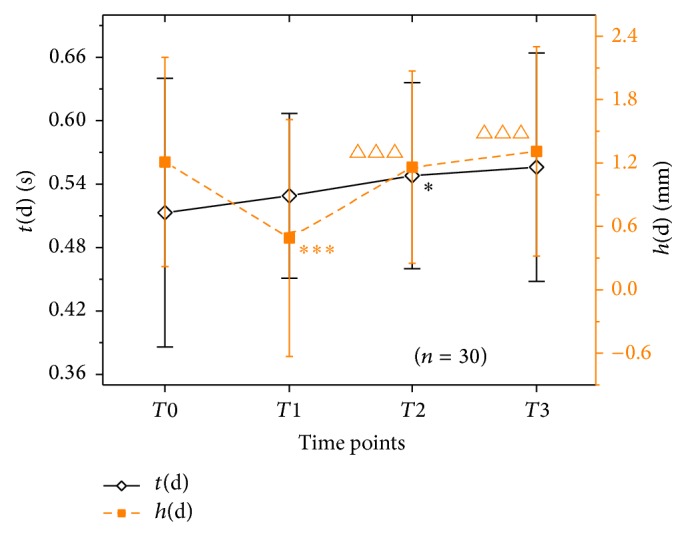
Pulsographic parameters during diastole in 30 PD patients at *T*0, *T*1, *T*2, and *T*3. Notes: ^*∗∗∗*^ *P* < 0.001 and ^*∗*^ *P* < 0.05, compared with *T*0; ^△△△^ *P* < 0.001, compared with *T*1.

As shown in Figure 4, from *T*0 to *T*1, time of the peak of dicrotic wave increased (*P* > 0.05) and amplitude of the peak of dicrotic wave (*h*(d)) significantly decreased (*P* < 0.001). From *T*1 to *T*3, *t*(d) increased (*P* > 0.05), while *h*(d) increased significantly (*P* < 0.001) firstly and then increased mildly (*P* > 0.05).

### 3.5. Changes in Heart Rate in PD Patients

**Figure 5:**
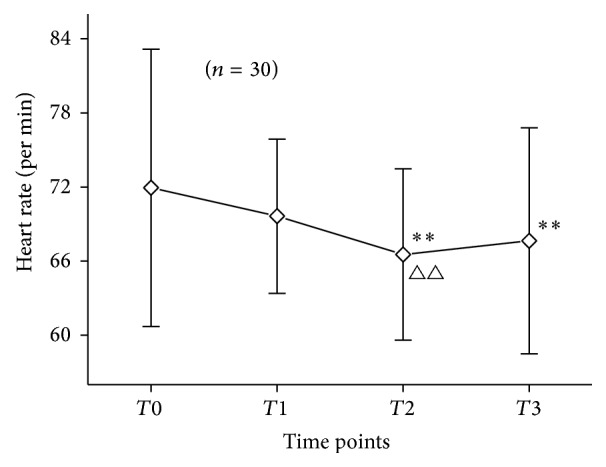
Changes in heart rate in 30 PD patients at *T*0, *T*1, *T*2, and *T*3. Notes: ^*∗∗*^ *P* < 0.01, compared with *T*0; ^△△^ *P* < 0.01, compared with *T*1.

Heart rate was 71.930 ± 11.22 times/min at *T*0 and decreased to 69.63 ± 6.24 times/min at *T*1 (*P* > 0.05). After acupuncture treatment, heart rate significantly decreased to 66.53 ± 6.94 times/min at *T*2 (*P* < 0.01) and increased to 67.63 ± 9.14 times/min at *T*3 (*P* > 0.05) (Figure 5).

### 3.6. Changes in Normalized-Based Time Parameters

**Figure 6:**
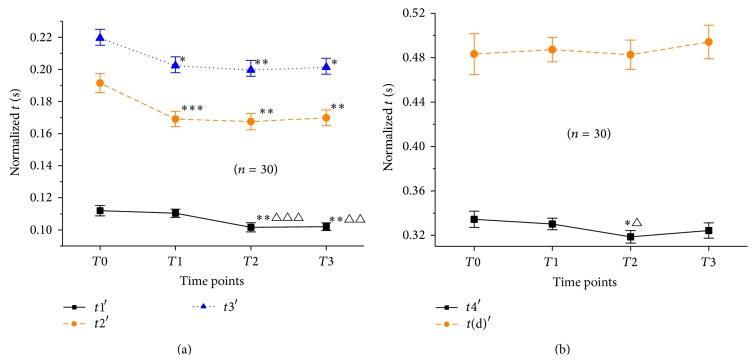
Changes in normalized-based time parameters at *T*0, *T*1, *T*2, and *T*3. Notes: (a) was changes in* t*1′,* t*2′, and* t*3′; (b) was changes in* t*4′ and* t*(d)′. ^*∗∗∗*^ *P* < 0.001, ^*∗∗*^ *P* < 0.01, and ^*∗*^ *P* < 0.05, compared with *T*0; ^△△△^ *P* < 0.001, ^△△^ *P* < 0.01, and ^△^ *P* < 0.05, compared with *T*1.

In view of the fact that time parameters will be effected by different heart rates, in this paper, time parameters of pulsograph at 4 time points were normalized at 75 times/min heart rate and named as* t*′. The comparison of* t*′ results after normalization is shown in Figure 6; compared with those at *T*0, there was no difference in* t*1′,* t*4′, and* t*(d)′ (*P* > 0.05) at *T*1. Meanwhile,* t*2′ and* t*3′ significantly decreased from *T*0 to *T*1 (*P* < 0.001, 0.05). From *T*1 to *T*3,* t*1′ and* t*4′ significantly decreased at first (*P* < 0.001) and then remained unchanged (*P* > 0.05). And there was no significant change in* t*2′,* t*3′, and* t*(d)′ from *T*1 to *T*3 (*P* > 0.05).

## 4. Discussion

Primary dysmenorrhea (PD) is defined as a cramp-like pain in the lower abdomen before or during menstruation without any identifiable pelvic pathology. Pain may be accompanied by lower back pain, nausea, vomiting, and diarrhea. PD is frequently found in young nullipara [17]. Modern medical science holds that the emergence of PD is related with many factors. Among them, temporary ischemia of myometrium and endometrium of the uterus plays a part. The ischemia may result from pressured intermuscular blood vessels induced by forceful contraction of uterine arteries and paroxysmal contraction of uterine smooth muscles [18]. Traditional Chinese Medicine holds that PD is often caused by* blood* stasis blocking the uterus. That is to say, during menstruation,* qi* and* blood* fail to flow freely. Pain arises as a result. Apart from pain, changes in movement of* qi* and* blood* would also affect* cunkou* pulse [19].

In this study, pulsographic parameters of* cunkou* pulse before dysmenorrhea and in obvious dysmenorrhea and after dysmenorrheal relieving in PD patients were investigated. Considering effect of different heart rates on the time parameters of pulsograph, in this experiment, the time parameters were normalized at the heart rate of 75 times/min. In so doing, we were more inclined to think that normalized time parameters could better reflect the effect of pain on time parameters in PD patients.

Changes in pulsographic parameters revealed almost opposite changing trends from before dysmenorrhea to before dysmenorrhea (from *T*0 to *T*1) and finally to after dysmenorrheal relieving (from *T*1 to *T*2). Although the effect of menstruation on pulsographic parameters cannot be excluded from *T*0 to *T*1, combining with the changes from *T*1 to *T*2 we can come to a preliminary conclusion that pain could cause increases in width of the upper 1/3 of main wave (*w*1), in amplitude of gorge of main wave (*h*2), and in amplitude of dicrotic notch (*h*4) and decreases in time of gorge of main wave (*t*2) and in amplitude of peak of dicrotic wave (*h*(d)).

Interestingly, we also noticed different changing trends from the commencement of acupuncture to 30 mins after acupuncture (from *T*1 to *T*2 and from *T*2 to *T*3). Part of parameters showed continuous similar changes. For example, *t*1 kept decreasing and *h*1, *t*4, *t*(d), and *h*(d) kept increasing. Some changes from *T*1 to *T*2 and from *T*2 to *T*3 were in opposite directions. For instance, *h*3 and *h*4 increased at first and then decreased, and *t*3 decreased at first and then increased. Still others changed significantly from *T*1 to *T*2 but no obvious changes from *T*2 to *T*3 were observed, such as *w*1 and *w*2, which decreased at first and then almost did not change. The possible reason for this phenomenon may be related to the negative feedback regulation which caused corresponding changes in the radial artery.

Previous researches show that each pulse is of specific pulsograph features. The changes in pulsographic parameters in our research could be interpreted with reference to wiry pulse, slippery pulse, and forceful pulse. The pulsograph of wiry pulse is characterized by higher prodicrotic wave, close to or fused to the main wave, manifesting as a broad single peak main wave, and higher dicrotic notch, and flat dicrotic wave. The pulsograph of slippery pulse is characterized by a higher double-humped main wave with big-slope ascending and descending branches, and a delayed prodicrotic wave, and a higher dicrotic wave. In addition, pulsograph of forceful pulse is characterized by a higher main wave [20]. In this paper, the pulsographs of patients with obvious pain manifested as wider upper 1/3 of main wave, and earlier gorge of main wave and higher prodicrotic wave, and higher dicrotic notch, and lower dicrotic wave, compared with pulsographs of those without pain. All these changes showed that pulsographs of patients with obvious pain resembled that of wiry pulse. However, when the pain is relieved, the above parameters change to the other way around. As the gorge of main wave gets higher and earlier, the dicrotic notch comes earlier while the time of dicrotic wave does not change obviously, which indicated that the slope of ascending and descending branches is elevated and there is a trend toward moving to a slippery pulse with a weakened pulse-power. This finding was consistent with the records in TCM that pain is often associated with wiry pulse.

As a pilot study we have to admit that there are some limitations in this study. Firstly, a limitation was the small number of enrolled patients and lack of control group. Without control group of healthy females, influence of menstruation on pulsograph could not be excluded. Secondly, the pain of PD cannot represent all kinds of pain owing to its localization and patient population particularity. Further studies on different types of pain influencing pulsograph are needed before we can know for sure how pain affects pulsographic parameters.

## 5. Conclusions

There are changes in pulsographic parameters, basically in opposite trends, in patients with PD when their pain occurs and when it is relieved. Further studies with control groups are needed to determine whether pulsographic parameters can be used as an objective indicator for pain changes in patients with primary dysmenorrhea.
